# Effect of Supplemental Parenteral Nutrition Versus Enteral Nutrition Alone on Clinical Outcomes in Critically Ill Adult Patients: A Systematic Review and Meta-Analysis of Randomized Controlled Trials

**DOI:** 10.3390/nu12102968

**Published:** 2020-09-28

**Authors:** Dalal J. Alsharif, Farah J. Alsharif, Ghadeer S. Aljuraiban, Mahmoud M. A. Abulmeaty

**Affiliations:** Department of Community Health Sciences, Clinical Nutrition Program, King Saud University, Riyadh 11362, Saudi Arabia; dalsharif@KSU.EDU.SA (D.J.A.); falsharif@KSU.EDU.SA (F.J.A.); galjuraiban@KSU.EDU.SA (G.S.A.)

**Keywords:** supplemental parenteral nutrition, intensive care, clinical outcomes

## Abstract

Enteral nutrition (EN) is considered the first feeding route for critically ill patients. However, adverse effects such as gastrointestinal complications limit its optimal provision, leading to inadequate energy and protein intake. We compared the clinical outcomes of supplemental parenteral nutrition added to EN (SPN + EN) and EN alone in critically ill adults. Electronic databases restricted to full-text randomized controlled trials available in the English language and published from January 1990 to January 2019 were searched. The risk of bias was evaluated using the Jadad scale, and the meta-analysis was conducted using the MedCalc software. A total of five studies were eligible for inclusion in the systematic review and meta-analysis. Compared to EN alone, SPN + EN decreased the risk of nosocomial infections (relative risk (RR) = 0.733, *p* = 0.032) and intensive care unit (ICU) mortality (RR = 0.569, *p* = 0.030). No significant differences were observed between SPN + EN and EN in the length of hospital stay, hospital mortality, length of ICU stay, and duration of mechanical ventilation. In conclusion, when enteral feeding fails to fulfill the energy requirements in critically ill adult patients, SPN may be beneficial as it helps in decreasing nosocomial infections and ICU mortality, in addition to increasing energy and protein intakes with no negative effects on other clinical outcomes.

## 1. Introduction

It is well established that enteral nutrition (EN) is considered the preferred feeding route for critically ill patients who cannot maintain a volitional intake of food [1,2,3,4]. Early EN (within 24–48 h of intensive care unit (ICU) admission) is recommended for patients with a functional gastrointestinal tract [5,6]. However, several complications limit the use of enteral feeding in critically ill patients, leading to suboptimal nutritional intake. These complications include diarrhea, vomiting, aspiration, and feeding interruptions [7,8,9]. A prospective multicenter study in 201 units from 26 countries with 3390 critically ill patients revealed that, on an average, only 61.2% and 57.6% of the prescribed calories and protein, respectively, were delivered to the patients [10]. A systematic review and meta-analysis of randomized controlled trials (RCTs) comparing the effects of EN and parenteral nutrition (PN) in critically ill patients reported no difference in the mortality rate, whereas EN alone decreased the incidence of bloodstream infections and the length of hospital stay, however, it did increase gastrointestinal complications [11]. Since malnutrition is prevalent among these patients, providing insufficient energy and protein may further worsen their already poor nutritional status [12,13].

Nonetheless, current guidelines for critically ill adults recommend early EN and the initiation of PN when nutritional goals are not met [6,14]. Supplementing EN with a partial dose of PN, also known as supplemental parenteral nutrition (SPN), is a strategy that might improve the nutritional intake of patients in the ICU [15]. However, according to a few previous meta-analyses on the effects of SPN and EN included trials that compared early versus late PN, some patients were initially administered PN, and EN was added later [16], and combining SPN and EN led to adverse outcomes and resulted in inconsistent conclusions. Previous systematic reviews and meta-analyses of RCTs comparing the effects of combined SPN and EN with EN alone in critically ill patients reported no difference in in-hospital mortality, length of ICU stay, and duration of ventilatory support. However, evidence of the effects of SPN on other outcomes, such as nutritional intake, is yet to be investigated [17]. Evidence on the use of SPN is limited, and further research is warranted [6].

Thus, the objective of this review and meta-analysis was to evaluate and compare the clinical outcomes of using SPN as a supplement to EN (SPN + EN) versus EN alone on ICU mortality, length of ICU stay, length of hospital stay, duration of mechanical ventilation, nosocomial infections, and protein and energy intakes in adult patients in the ICU. For an accurate assessment of the effects of SPN, SPN + EN was defined as either EN with SPN provided to the patient on day one in the ICU or SPN provided to patients already receiving EN; therefore, trials that provided EN to patients who previously received significant calories from PN were excluded.

## 2. Materials and Methods

### 2.1. Protocol and Registration

We followed the Cochrane Handbook for Systematic Reviews of Intervention in addition to the PRISMA guidelines [18,19]. The protocol for this systematic review and meta-analysis was registered in PROSPERO (CRD42019121888) [20].

### 2.2. Eligibility Criteria

Type of studies: Full-text RCT articles available in the English language published from January 1990 to January 2019 were considered.

Type of participants: Studies on adults (≥16 years old) who were critically ill or admitted to the ICU were included.

Types of interventions: Studies comparing the effects of SPN + EN (only when SPN is added with or after EN) versus EN alone were included.

Outcomes:

Primary Outcomes

ICU mortality, length of ICU stay, length of hospital stay, duration of mechanical ventilation, and nosocomial infections

Secondary Outcomes

The effects of SPN + EN on protein and energy intakes were assessed as secondary outcomes and were included when they were available in the same studies along with the clinical outcomes.

### 2.3. Search Strategy

The electronic databases PubMed, EMBASE, Google Scholar, Scopus, and Cochrane Central Register of Controlled Trials were searched systematically for eligible studies during the period between September 2019 and January 2020. Other sources of grey literature such as clinicaltrials.gov, ProQuest, and European Society for Clinical Nutrition and Metabolism (ESPEN) congress abstracts, were also searched for possibly related articles. The search was restricted to full-text articles available in the English language that were published from January 1990 to January 2019. The following search terms were used: “supplemental parenteral nutrition”, “combined enteral and parenteral nutrition,” “enteral nutrition”, “ICU”, “critical care”, “critically ill adult”, randomized controlled trial” and their derivatives. Each term was used separately and in combination with other terms (see Appendix A for the search strategy). Any possibly related citations were added to the citation manager (Mendeley) for further abstract screening. Filters were applied while searching to limit the date and language of publication. The process was performed separately by two independent researchers (D.J.A. and F.J.A.) and was repeated for all databases.

### 2.4. Study Selection

While screening abstracts, all studies involving SPN in adult patients were included for a full-text assessment. Full-text articles were evaluated by two researchers (M.M.A.A. and G.S.A.) for conformance with the eligibility criteria and were checked by a third researcher when necessary (M.M.A.A.).

### 2.5. Data Collection Process

Two researchers (D.J.A. and F.J.A.) were involved in extracting the data independently, which were then checked by a third researcher (M.M.A.A. or G.S.A.) for any missing information. Discrepancies were resolved based on maximum votes (three out of four votes). Investigators used two pre-set tables to extract data. While the first table was used for extracting the characteristics of studies, the second table was used for extracting the outcomes, including ICU mortality, length of ICU stay, length of hospital stay, duration of mechanical ventilation, hospital-acquired infections, and energy and protein intakes. The authors of the corresponding studies were contacted through email to confirm the data when needed.

### 2.6. Quality Assessment

The risk of bias was examined independently by two researchers (D.J.A. and F.J.A.) using the Jadad scale, a three-criteria appraisal form that focuses on randomization, blinding, and accounts for all patients [21]. The scale had a maximum score of five and was applied to all studies meeting the eligibility criteria. Studies were excluded if they scored less than three on the Jadad Scale.

### 2.7. Statistical Analysis

The systematic review analysis was carried out by applying meta-analysis for both continuous outcome variables (length of ICU stay, length of hospital stay, duration of mechanical ventilation, energy intake, and protein intake) and categorical outcome variables (ICU mortality and infection). Means, standard deviations, frequencies, and percentages were used to describe the outcome variables. Standardized mean difference (SMD) was calculated as a summary pooled statistic using the cutoffs as recommended by Cohen (pooled effect of 0.2, small; 0.5, medium; and, 0.8 and above, large). The statistical significance of SMD was assessed using Student’s t-test. For categorical outcome variables, pooled relative risk (RR) was used, where pooled RR < 1 shows a reduction in risk, and pooled RR > 1 shows an increase in risk. To identify heterogeneity in the pooled data, Cochran’s Q test (weighted sum of squares on a standardized scale) was used. Additionally, I^2^ was used to indicate the percentage of total variation across the studies included in the meta-analysis. A cutoff value of I^2^ > 50% was applied to rule out higher levels of unexplained variation in the effect sizes. Pooled estimates were obtained using both the fixed effect and random effect models. Statistical significance and precision of estimates were reported using *p* ≤ 0.05 and 95% confidence intervals. Forest plots were used to report the results (overall effect using both fixed and random effect models) of the studies included in the meta-analysis. All analyses were performed using MedCalc for Windows version 15.0 (MedCalc Software, Ostend, Belgium) [22].

## 3. Results

### 3.1. Study Identification and Selection

**Figure 1:**
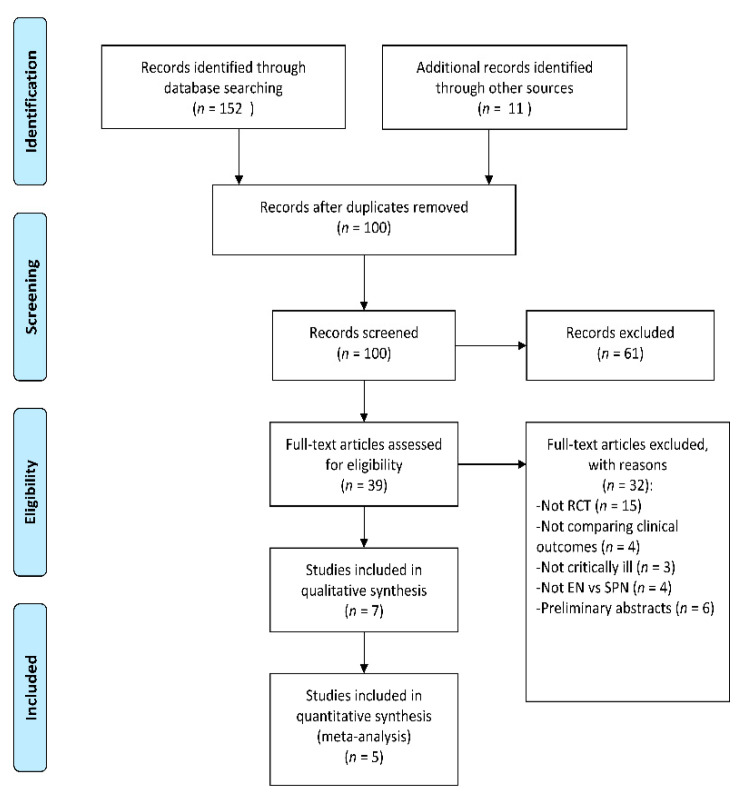
Flow diagram of the search strategy. Randomized controlled trials (RCT), Supplemental parenteral nutrition (SPN), Enteral nutrition (EN).

The search strategy yielded a total of 163 articles (Figure 1), out of which 63 duplicate articles were removed. After screening the abstracts, 61 articles were omitted because they were either reviews or unrelated articles such as those dealing with pediatric patients or costs of SPN. After assessing the full-text articles, an additional 32 were excluded either because they were not RCTs available in full-text or they did not (a) include critically ill patients, (b) compare EN with SPN + EN, or (c) compare clinical outcomes. Details about the excluded RCTs are available in Table S1. Seven articles met the eligibility criteria and were assessed for the risk of bias using the Jadad Scale (Table S2). Of these, five articles scored three or more on the Jadad scale and were included in the systematic review and meta-analysis. The characteristics of the included studies are available in Table 1.

### 3.2. Effect of SPN on Clinical Outcomes in Critically Ill Patients

#### 3.2.1. ICU Mortality

**Figure 2:**
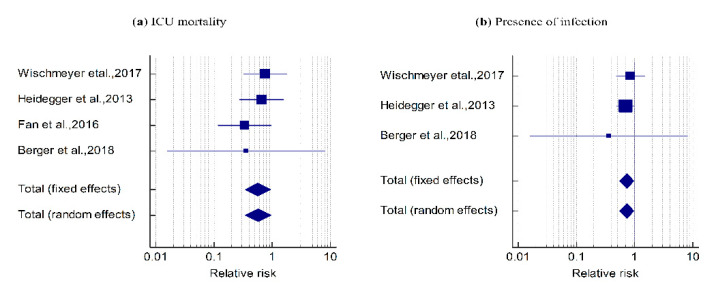
Forest plot showing the effect of the SPN + EN on (**a**) ICU mortality: pooled RR = 0.569, *z* = −2.165, *p* = 0.030. The Cohran’s Q was not statistically significant (Q = 1.641, *p* = 0.650) and I^2^ = 0.00%. (**b**) The presence of infection events: pooled RR = 0.733, *z* = −2.145, *p* = 0.032. Q = 0.551, *p* = 0.759 and I^2^ value = 0.00%.

The mortality events related to SPN + EN were lower than those related to EN (pooled RR = 0.569, *z* = −2.165, *p* = 0.030); that is, the risk of ICU mortality was reduced by 0.43 (43.1%) with SPN + EN as compared to EN alone (Table 2 and Figure 2a). Cochran’s Q value was not statistically significant (Q = 1.641, *p* = 0.650), and the I^2^ value (0.00%) revealed homogeneity across the four studies.

#### 3.2.2. Presence of Infection

In three studies, the presence of infection events was lower with SPN + EN when compared to EN alone (pooled RR = 0.733, *z* = −2.145, *p* = 0.032), indicating that the risk of occurrence of infection was reduced by 0.267 (26.7%) with SPN + EN as compared to EN alone (Table 2 and Figure 2b). Cochran’s Q value was not statistically significant (Q = 0.551, *p* = 0.759) and the I^2^ value (0.00%) showed homogeneity across the three studies.

#### 3.2.3. Length of Hospital Stay

**Figure 3:**
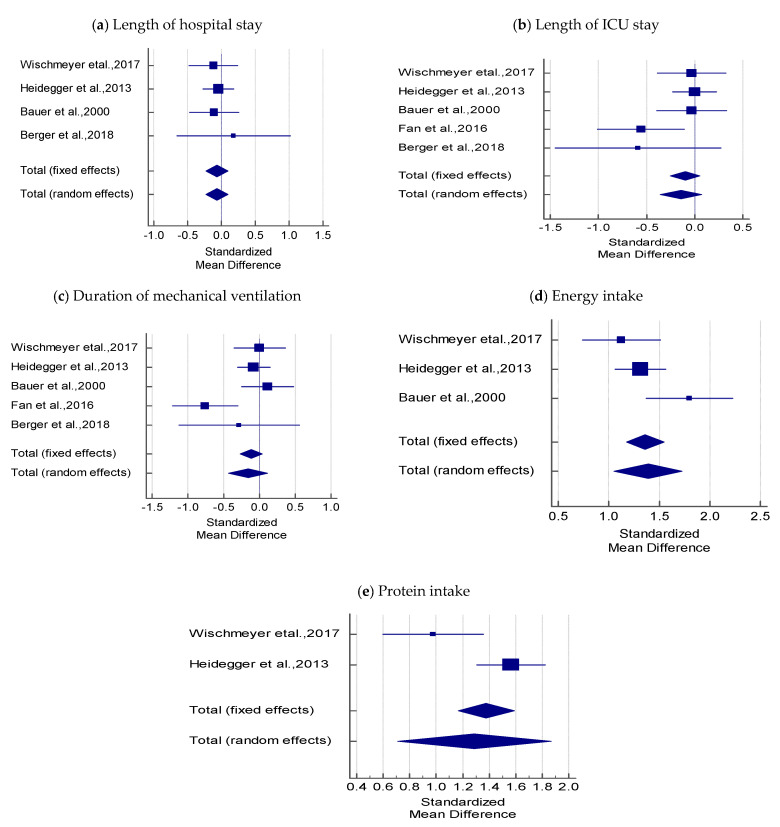
Forest plots comparing the effect of SPN + EN on; (**a**) Hospital stay, (**b**) Length of ICU stay, (**c**) Duration of mechanical ventilation, (**d**) Energy intake, (**e**) Protein intake.

In four studies, there was no statistical difference in hospital stay between SPN + EN and EN using fixed and random effects models (SMD = −0.083 *t* = −0.752, *p* = 0.452; Table 3 and Figure 3a). The overall effect was very small (−0.083 < 0.2). Cochran’s Q value was not statistically significant (0.5373, *p* = 0.911), and the I^2^ value (0.00%) indicated no heterogeneity among the four studies.

#### 3.2.4. Length of ICU Stay

A fixed-effect criterion was considered because both Cohran’s Q (6.545) and I^2^ values (38.88%) were not significantly high. In five studies, the pooled estimate showed no significant difference in the mean length of ICU stay between the SPN + EN and EN groups (SMD = −0.100, *t* = −1.278; *p* = 0.202), and the overall effect was very small (−0.100 < 0.2; Table 3 and Figure 3b).

#### 3.2.5. Duration of Mechanical Ventilation

The pooled SMD using the random-effects model showed no significant difference in the mean values of duration of mechanical ventilation between SPN + EN and EN (SMD = −0.159, *t* = −1.139, *p* = 0.255); the overall effect was very small (−0.159 < 0.2). Cochran’s Q value was statistically significant (Q = 10.195, *p* = 0.037), and the I^2^ value (60.77%) was high, indicating heterogeneity across the five studies (Table 3 and Figure 3c).

### 3.3. Effect of SPN on Energy and Protein Intake in Critically Ill Patients

#### 3.3.1. Energy Intake

The pooled SMD by the random-effects model was used to infer that SPN + EN had higher mean values of energy intake when compared with EN (SMD = 1.391, *t* = 8.097, *p* < 0.001). The overall effect was 1.391 > 0.8. Cochran’ s Q value was statistically significant (Q = 5.719, *p* = 0.057) and the I^2^ value (65.03%) was high, indicating heterogeneity among the three studies (Table 3 and Figure 3d).

#### 3.3.2. Protein Intake

Cochran’ s Q value (Q = 6.517, *p* = 0.011) was statistically significant and the I^2^ value (84.66%) was high, indicating heterogeneity across the two studies (Table 3 and Figure 3e). Hence, the pooled SMD using the random-effects model revealed that the mean protein intake was significantly higher in SPN + EN than in EN (SMD = 1.287, *t* = 4.371, *p* < 0.001). The overall effect was large (1.287 > 0.8).

## 4. Discussion

The current meta-analysis revealed that compared to EN alone, SPN + EN was not associated with an increase in the lengths of hospital stay, ICU stay, and mechanical ventilation (Figure 3a–c). However, SPN + EN was associated with a decrease in ICU mortality and hospital-acquired infections (Figure 2) without adversely affecting other clinical outcomes. Moreover, combining EN with SPN improved the protein and energy intakes in critically ill adult patients (Figure 3d,e). These findings demonstrate the benefits of SPN + EN in situations where enteral feeding alone fails to fulfill the energy requirements of critically ill patients.

We addressed the methodological limitations of previous meta-analyses of RCTs that compared the effects of SPN + EN with EN alone [17,28]. For example, we included only high-quality RCTs and excluded RCTs scoring less than three on the Jadad Scale. In addition, our meta-analysis only included studies comparing SPN + EN with EN alone, while previous meta-analyses included studies comparing early and late PN combined with EN [17,28]. For example, in one study that included in both the analyses, the Early versus Late Parenteral Nutrition in Critically ill adults (EPaNIC) study, early PN administration (within the first day of ICU admission) was associated with an increased risk of nosocomial infections as well as longer durations of mechanical ventilation and hospital stay [16]. However, the EPaNIC study differed from studies included in the current meta-analysis in that both the intervention and standard groups received PN to supplement insufficient energy intake from EN. The intervention group received PN early whereas in the standard group, PN was initiated after day eight. In the intervention group, the patients received 800 kcal from glucose infusion before starting EN on day three. In the intervention group, some patients did not receive SPN when EN was sufficient [16]. Our meta-analysis included studies where PN was initiated along with EN within 48–72 h of hospital admission [23,25,27], while in two studies, PN was initiated on day 4 [24,26].

In contrast with the recommendations of the American Society of Parenteral and Enteral Nutrition, our meta-analysis revealed that SPN + EN could be beneficial in critically ill patients for increasing their protein and energy intakes as well as decreasing the risk of ICU mortality and nosocomial infections with no adverse effects on other clinical outcomes even when initiated before day eight [5]. This recommendation is different from the recent ESPEN guidelines [6], where SPN is recommended for critically ill adults and its efficacy should be weighted depending on the case. Several studies have shown that the administration of adequate energy in critically ill patients improved clinical outcomes, which may explain the decrease in the rates of nosocomial infections associated with SPN + EN [29,30,31]. The SPN Swiss study by Pradelli et al. revealed that every 1000 kcal reduction in the cumulative energy deficit was linked to a 10% decrease in the risk of nosocomial infections [32]. In addition, the medical savings per avoided infection were CHF 63,048 [32]. Thus, SPN + EN providing adequate levels of protein and energy is also a cost-saving strategy that reduces expenses associated with infections.

The small number of studies (*n* = 5) is a limitation of the current meta-analysis. This was because only a few RCTs have compared the effects of SPN + EN to EN alone [23,24,25,26,27,33,34]. Therefore, more studies are needed to confirm the results of the current systematic review. Additionally, the included studies had different categories of ICU patients (burn, trauma, and others), and the responses to the interventions were different in each category. Moreover, several cofounding factors that interfere with the effects of SPN + EN and could have influenced the results of the analysis were not accounted for. These include the type of enteral formula used, the form of lipids used in the PN solution, and the equations used to estimate the energy requirement. Energy targets in the included studies ranged from to 20–30 kcal/kg using either the actual, ideal, or adjusted body weight [23,25,26,27]. The energy target was validated by indirect calorimetry in only one study [24]. Although the target energy intake seems similar between studies, it was not individualized based on each patient’s needs. Individualization of energy based on indirect calorimetry is recommended to avoid overfeeding [6].

Despite these limitations, the current meta-analysis is of clinical importance because it highlights the potential benefits of SPN + EN, especially in cases where EN alone is insufficient. A few trials have reported that PN is not associated with increased mortality [35,36]. In the CALORIES randomized controlled multicenter trial, no significant differences in infectious complications and 30-day and 90-day mortality were reported between patients on EN or PN [35]. In addition,, in the NUTRIREA-2 study, there was no significant difference between the two groups with respect to mortality (measured by day 28) and ICU-acquired infections [36]. Furthermore, EN was associated with a higher risk of digestive complications [36]. Thus, the benefits of adding PN to EN might outweigh its risk when added at the right time and in the right amount. EN should be provided during the first 24–48 h of admission, but if it does not fulfill the nutritional energy requirement by day four, SPN should be considered.

## 5. Conclusions

When EN fails to fulfill the energy requirements in critically ill patients, SPN might be considered as it helps in (a) increasing the energy and protein intake and (b) decreasing nosocomial infections and ICU mortality without a significant increase in in-hospital mortality and the lengths of hospital stay, ICU stay, and mechanical ventilation. To obtain the maximum benefits from SPN, it should be delayed until at least day four after the initiation of EN to allow EN to progress sufficiently and decrease the amount of SPN needed.

## Figures and Tables

**Table 1:** Characteristics of the included studies.

Author	Year	Design	*N*	Settings	Main Diagnosis	APACHE II Score	Duration	Energy and/or Protein Intake	Inclusion Criteria	Interventions
Bauer et al. [23]	2000	RCT	120 SPN + EN: 60 EN: 60	Single-center—two intensive care units (medical and surgical)	Multiple trauma, respiratory failure, stroke, sepsis, coronary artery disease, poisoning, renal failure, and gastrointestinal bleeding.	SAPS II SPN + EN: 43 ± 14 EN: 41 ± 13	7 days	SPN + EN: 9.9 ± 3.1 Kcal/kg/d from SPN and 14.8 ± 4.6 Kcal/kg/d from EN (total = 24.6 ± 4.9 Kcal/kg/d) SPN + EN: 13.2 ± 4.3 Kcal/kg/d from EN and 1.1 ± 0.3 Kcal/kg/d from SPN (total = 14.6 ± 6.5 Kcal/kg/d)	Adult patients in ICU aged ≥ 18 years expected to receive progressive enteral feeding for more than 2 days, to receive less than 20 kcal/kg/day for more than 2 days and stay in ICU for more than 2 days	Patients were randomly assigned to receive either parenteral plus enteral nutrition or enteral nutrition plus placebo for 4–7 days after starting nutritional support. The energy target was 25 kcal/kg
Berger et al. [24]	2018	RCT	28 SPN + EN: 11 EN: 12	Single Center (multidisciplinary ICU)	Medical/surgical patients	SPN + EN: 25 (17–26) EN: 23 (19.2–27.8)	5 days	SPN + EN: average total energy intake = 24.3 Kcal/kg/d and protein = 1.16 g/kg/d EN: average total energy intake = 16.1 Kcal/kg/d and protein = 0.76 g/kg/d	Adults in ICU, mechanically ventilated patients with a functional gut, who received < 60% of their energy requirements by day 3	Patients were randomly assigned to EN or SPN + EN with the target energy requirements validated by indirect calorimetry
Fan et al. [25]	2016	RCT	120 SPN + EN: 40 EN: 40 PN: 40	Single-center (Neurological intensive care unit)	Severe traumatic brain injury	N/A N/A	20 days	SPN + EN: 1500 Kcal/d from EN and remaining amount until the target of (25–30 kcal/kg/day) from SPN EN: 2250 Kcal/d PN: 25–30 Kcal/kg/d of PN solution; ratio 2:1 for carbohydrates to lipids and 100:1 for kcal to nitrogen	Adults admitted to the neurological intensive care unit with severe traumatic brain injury diagnosis with Glasgow Coma Scale of 6–8 and Nutrition Risk Screening ≥ 3	Patients were randomized into three groups: EN, PN, EN + PN based on the sequence of their hospital record numbers. All patients were provided 25–30 kcal/kg of nutritional requirements
Heidegger et al. [26]	2013	RCT	305 SPN + EN: 153 EN: 152	Two-center (medical and surgical ICU of two tertiary care hospitals)	Shock, neurological, cardiac surgery, polytrauma, pneumonia, cardiac arrest, respiratory failure, myocardial infarction, acute pancreatitis, and liver failure	SPN + EN 22 ± 7 EN 23 ± 7	5 days	SPN + EN: 100% of the target (1892 Kcal/d and 81 g protein/d); 75% as EN and 25% as SPN EN: the target (1836 Kcal/d and 80 g protein/d); 80% as EN and non-nutritional fluids	Adults with functional gastrointestinal tract and expected ICU stay exceeding five days, expected survival rate exceeding 1 week and had received less than 60% of their energy requirement from EN on the third day of ICU admission	Patients were randomly assigned to receive EN or SPN + EN. Energy targets were calculated using indirect calorimetry or by multiplying 25–30 kcal per kg of ideal body weight
Wischmeyer et al. [27]	2017	RCT	125 SPN + EN: 52 EN: 73	Multicenter (11 centers across four countries)	Respiratory, sepsis, gastrointestinal, neurologic, trauma, metabolic, cardiovascular/vascular hematologic	SPN + EN 20.5 ± 6.4 EN 20.8 ± 7.2	7 days	SPN + EN: 95 ± 13% of the calorie target, and 82 ± 19% of the protein target EN: 69 ± 28% of the calorie target, and 64 ± 26% of the protein target	Mechanically ventilated adult patients aged > 18 years with BMI < 25 or > 35, with acute respiratory failure, who received EN or were to be started on EN within 48 h of ICU admission	Patients were randomized to receive EN alone or SPN + EN to reach their full nutritional requirements within 7 days after randomization. The energy target was 20–25 kcal/kg based on BMI

Randomized controlled trials (RCT), Supplemental parenteral nutrition (SPN), Enteral nutrition (EN), Acute Physiology and Chronic Health Evaluation II (APACHE II), Simplified Acute Physiology Score II (SAPS II), Intensive care unit (ICU), Body mass index (BMI), Not applicable (N/A).

**Table 2:** Meta-Analysis for the outcome variables: ICU mortality and presence of infection ^1,2^.

Study	SPN + EN	EN	Relative Risk	95% CI	z-Value	*p*-Value	Weight (%)	
	No. of Events/Total	No. of Events/Total					Fixed	Random
**ICU Mortality ^1^**								
Wischmeyer et al., 2017	7/52	13/73	0.756	0.324 to 1.764			37.16	37.16
Heidegger et al., 2013	8/153	12/152	0.662	0.279 to 1.575			35.57	35.57
Fan et al., 2016	4/40	12/40	0.333	0.117 to 0.946			24.51	24.51
Berger et al., 2018	0/11	1/12	0.361	0.016 to 8.040			2.77	2.77
Total (fixed effects)	19/256	38/277	0.569	0.342 to 0.948	−2.165	0.030	100.00	100.00
Total (random effects)	19/256	38/277	0.578	0.345 to 0.969	−2.080	0.038	100.00	100.00
**Presence of Infection ^2^**								
Wischmeyer et al., 2017	14/52	23/73	0.855	0.488 to 1.498			25.59	25.59
Heidegger et al., 2013	41/153	58/152	0.702	0.504 to 0.978			73.57	73.57
Berger et al., 2018	0/11	1/12	0.361	0.016 to 8.040			0.84	0.84
Total (fixed effects)	55/216	82/237	0.733	0.552 to 0.974	−2.145	0.032	100.00	100.00
Total (random effects)	55/216	82/237	0.734	0.553 to 0.975	−2.132	0.033	100.00	100.00

^1^ Test for heterogeneity: Q = 1.641; df = 3; *p* = 0.650; I2 (inconsistency) = 0.00%; 95% CI for I2 = 0.00% to 76.40%. ^2^ Test for heterogeneity: Q = 0.551; df = 2; *p* = 0.759; I2 (inconsistency) = 0.00%; 95% CI for I2 = 0.00% to 87.83%.

**Table 3:** Meta-Analysis for the outcome variables: length of hospital stay, length of ICU stay, duration of mechanical ventilation, energy intake, and protein intake.

Study	SPN + EN		EN		SMD	95% CI	t-Value	*p*-Value	Weight (%)	
	N1	Mean (SD)	N2	Mean (SD)					Fixed	Random
**Length of hospital stay**										
Wischmeyer et al., 2017	52	26.0 (5.2)	73	26.7 (6.4)	−0.117	−0.475 to 0.240			21.34	21.34
Heidegger et al., 2013	153	31 (23)	152	32 (23)	−0.043	−0.268 to 0.181			53.28	53.28
Bauer et al., 2000	60	31.2 (18.5)	60	33.7 (27.7)	−0.105	−0.465 to 0.254			21.10	21.10
Berger et al., 2018	11	41.8 (8.5)	12	39.8 (12.4)	0.180	−0.659 to 1.018			4.28	4.28
Total (fixed effects)	276		297		0.083	−0.226 to 0.101	−0.752	0.452	100.00	100.00
Total (random effects)	276		297		0.083	−0.226 to 0.101	−0.752	0.452	100.00	100.00
Test for heterogeneity: Q = 0.5373; df = 3; *p* = 0.911; I^2^ = 0.00% (95% CI: 0.00% to 27.91%)										
**Length of ICU stay**										
Wischmeyer et al., 2017	52	12.9 (2.9)	73	13.1 (2.8)	−0.031	−0.388 to 0.326			18.83	21.85
Heidegger et al., 2013	153	13 (10)	152	13 (11)	0.000	−0.225 to 0.225			46.93	33.83
Bauer et al., 2000	60	16.9 (11.8)	60	17.3 (12.8)	−0.032	−0.392 to 0.327			18.61	21.70
Fan et al., 2016	40	27.6 (7.5)	40	31.4 (5.9)	−0.556	−1.01 to −0.107			12.02	16.36
Berger et al., 2018	11	13.6 (2.2)	12	15.9 (5.1)	−0.589	−1.444 to 0.267			3.62	6.26
Total (fixed effects)	316		337		−0.100	−0.254 to 0.054	−1.278	0.202	100.00	100.00
Total (random effects)	316		337		−0.142	−0.357 to 0.074	−1.293	0.197	100.00	100.00
Test for heterogeneity: Q = 6.545; df = 4; *p* = 0.162; I^2^ = 38.88% (95% CI: 0.00% to 77.35%)										
**Duration of mechanical ventilation**										
Wischmeyer et al., 2017	52	8.5 (9)	73	8.5 (2.6)	0.003	−0.354 to 0.360			18.89	22.25
Heidegger et al., 2013	153	153 (163)	152	166 (160)	−0.080	−0.305 to 0.145			47.04	28.62
Bauer et al., 2000	60	11 (9)	60	10 (8)	0.117	−0.243 to 0.476			18.64	22.14
Fan et al., 2016	40	8.4 (4.7)	40	12.6 (6.1)	−0.759	−1.215 to −0.302			11.67	18.09
Berger et al., 2018	11	10.5 (4.1)	12	11.5 (2.4)	−0.284	−1.125 to 0.558			3.76	8.90
Total (fixed effects)	316		337		−0.115	−0.269 to 0.039	−1.462	0.144	100.00	100.00
Total (random effects)	316		337		−0.159	−0.433 to 0.115	−1.139	0.255	100.00	100.00
Test for heterogeneity: Q = 10.195; df = 4; *p* = 0.037; I^2^ = 60.77% (95% CI: 0.00% to 85.29%)										
**Energy intake**										
Wischmeyer et al., 2017	52	95 (13)	73	69 (28)	1.124	0.740 to 1.507			23.93	31.13
Heidegger et al., 2013	153	28 (5)	152	20 (7)	1.313	1.065 to 1.561			56.66	40.37
Bauer et al., 2000	60	24.6 (4.9)	60	14.2 (6.5)	1.795	1.369 to 2.222			19.41	28.50
Total (fixed effects)	265		285		1.361	1.175 to 1.547	14.352	<0.001	100.00	100.00
Total (random effects)	265		285		1.391	1.054 to 1.729	8.097	<0.001	100.00	100.00
Test for heterogeneity: Q = 5.719; df = 2; *p* = 0.057; I^2^ = 65.03% (95% CI: 0.00% to 89.96%)										
**Protein intake**										
Wischmeyer et al., 2017	52	86(16)	73	64 (26)	0.976	0.599 to 1.353			31.96	47.23
Heidegger et al., 2013	153	1.2(0.2)	152	0.8 (0.3)	1.566	1.309 to 1.823			68.04	52.77
Total (fixed effects)	205		225		1.377	1.166 to 1.589	12.782	<0.001	100.00	100.00
Total (random effects)	205		225		1.287	0.708 to 1.866	4.371	<0.001	100.00	100.00
Test for heterogeneity: Q = 6.517; df = 1; *p* = 0.011; I^2^ = 84.66% (95% CI: 37.31% to 96.24%)										

Standardized mean difference (SMD).

## Supplementary Materials

The following are available online at https://www.mdpi.com/2072-6643/12/10/2968/s1, Table S1. Excluded Studies [15,16,33,34,37,38,39,40,41,42,43,44,45,46,47,48,49,50,51,52,53,54,55,56,57,58,59,60,61,62,63,64,65,66], and Table S2. Quality assessment of the selected studies by the Jadad Scale.

## Appendix A. Pubmed and Embase Search Strategy

Pubmed search strategy

#1 intensive care units [MeSH Terms] OR critical care [Title/Abstract] OR critical illness [Title/Abstract] OR critically ill [Title/Abstract] OR intensive care [Title/Abstract] OR ICU [Title/Abstract]

#2 parenteral nutrition [MeSH Terms] OR parenteral nutrition [Title/Abstract] OR parenteral, nutrition [Title/Abstract] OR combined enteral and parenteral nutrition [Title/Abstract] OR supplemental parenteral nutrition [Title/Abstract] OR PN [Title/Abstract]

#3 enteral nutrition [MeSH Terms] OR enteral nutrition [Title/Abstract] OR enteral, nutrition [Title/Abstract] OR tube feeding [Title/Abstract] OR enteral feeding [Title/Abstract] OR EN [Title/Abstract]

#4 randomized controlled trial [MeSH Terms] OR randomized controlled trial [Title/Abstract] OR clinical trial [Title/Abstract] OR study [Title/Abstract]

#5 humans [MeSH Terms] AND adult [MeSH Terms] NOT animals [MeSH Terms]

#6 #1 AND #2 AND #3 AND #4 AND #5

For Embase, the search strategy was:

#1 ‘critically ill patient’/exp OR ‘intensive care unit’/exp OR ‘intensive care’/exp OR ‘critical illness’/exp

#2 ‘critically ill’:ti,ab,kw OR ‘intensive care unit’:ti,ab,kw OR ‘icu’:ti,ab,kw OR ‘critical illness’:ti,ab,kw

#3 #1 OR #2

#4 ‘enteric feeding’/exp OR ‘enteric feeding’:ti,ab,kw OR ‘enteral, nutrition’:ti,ab,kw

#5 ‘parenteral nutrition’/exp OR ‘parenteral nutrition’:ti,ab,kw OR ‘parenteral, nutrition’:ti,ab,kw OR ‘combined enteral and parenteral nutrition’:ti,ab,kw OR ‘supplemental parenteral nutrition’:ti,ab,kw

#6 #4 AND #5

#7 ‘crossover-procedure’/exp OR ‘double-blind procedure’/exp OR ‘randomized controlled trial’/exp OR ‘single-blind procedure’/exp

#8 ‘random*’:ti,ab,kw OR ‘blind*’:ti,ab,kw OR ‘placebo’:ti,ab,kw

#9 7 OR 8

#10 [adult]/lim OR [middle aged]/lim OR [aged]/lim OR [very elderly]/lim)

#11 [humans]/lim

#12 #11 AND #12

#13 #3 AND #6 AND #9 AND #12
