# Supplementary material for: Effect of Supplemental Parenteral Nutrition Versus Enteral Nutrition Alone on Clinical Outcomes in Critically Ill Adult Patients: A Systematic Review and Meta-Analysis of Randomized Controlled Trials
Source: Nutrients. 2020 Sep 28;12(10):2968. doi: 10.3390/nu12102968 (PMC7601814; doi:10.3390/nu12102968)
Supplement: Supplementary file 1 [file nutrients-12-02968-s001.pdf]

Table S1. Excluded Studies.

Study	Reason for Exclusion
Ridley et al.,2018 **[15]**	Not EN alone vs. SPN
Casaer et al., 2011**[16]**	The study compared early vs. Late SPN
Dunham et al.,1994 **[33]**	Low Jadad score
Abrishami et al., 2010 **[34]**	Low Jadad Score
Allingstrup et al., 2017 **[37]**	The study compared the effect of early goal directed nutrition versus standard care and included early and late initiation of SPN
Berger et al., 2011 **[38]**	Preliminary abstract
Berger et al., 2018 **[39]**	Preliminary abstract
Caccialanza et al., 2018 **[40]**	Not critically ill patients
Chao et al., 2017 **[41]**	Not RCT and not conducted on critically ill or ICU patients
Deegan et al., 1999 **[42]**	Not RCT (retrospective study)
Elke et al., 2008 **[43]**	Not RCT (prospective observational study)
El-Sayed et al., 2015 **[44]**	The study did not assess the effect on clinical outcomes
Gavri et al., 2016 **[45]**	Not RCT (observational study)
Graf et al., 2012 **[46]**	Preliminary abstract
Heidegger et al., 2011 **[47]**	Preliminary abstract
Heyland et al., 2010 **[48]**	Not RCT
Hsu et al., 2012 **[49]**	Not RCT (retrospective study)
Huang et al., 2000 **[50]**	Not RCT (nutritional support was based on physician's concerns)
Kutsogiannis et al., 2011 **[51]**	Not RCT (observational study)
Lidder et al., 2010 **[52]**	Not conducted on critically ill patients
Mazaherpur et al., 2016 **[53]**	The study did not assess the effect on clinical outcomes
Nagata et al., 2009 **[54]**	Not conducted on critically ill or ICU patients
Nataloni et al., 1999 **[55]**	The study did not assess the effect on clinical outcomes
Oertel et al., 2009 **[56]**	Not RCT
Probst et al., 2016 **[57]**	Not RCT (retrospective cohort study)
Sena et al., 2008 **[58]**	Not RCT (retrospective cohort study)
Singh et al., 2012 **[59]**	Not RCT and not critically ill or ICU patients
Theilla et al., 2018 **[60]**	Not RCT
Thibault et al., 2009 **[61]**	Preliminary abstract
Thibault et al., 2010 **[62]**	Preliminary abstract
Titova et al., 2014 **[63]**	Not assessing clinical outcomes
Vallejo et al., 2017 **[64]**	Not RCT (retrospective observational study)
Qhi et al., 2016 **[65]**	Not RCT
Zhu et al., 2013 **[66]**	Not compared to EN alone

Table S1. Quality assessment of the selected studies by the Jadad Scale.

Study	Randomization	Blinding	An Account of withdrawals and dropouts	Jaded Score
Fan et al., 2016 **[24]**	2	0	1	3
Wischmeyer et al., 2017 **[25]**	2	0	1	3
Bauer et al., 2000 **[26]**	2	1	1	4
Heidegger et al., 2013 **[27]**	2	0	1	3
Berger et al., 2018 **[28]**	2	0	1	3
Dunham et al., 1994 **[33]**	1	0	1	2
Abrishami et al., 2010 **[34]**	1	0	1	2
